# No difference in patient reported outcomes, laxity, and failure rate after revision ACL reconstruction with quadriceps tendon compared to hamstring tendon graft: a systematic review and meta-analysis

**DOI:** 10.1007/s00167-023-07380-5

**Published:** 2023-03-24

**Authors:** Amit Meena, Stefano Di Paolo, Alberto Grassi, Akshya Raj, Luca Farinelli, Christian Hoser, Sachin Tapasvi, Stefano Zaffagnini, Christian Fink

**Affiliations:** 1 Gelenkpunkt-Sports and Joint Surgery, FIFA Medical Centre of Excellence, Olympiastraße 39, 6020 Innsbruck, Austria; 2 Research Unit for Orthopedic Sports Medicine and Injury Prevention (OSMI), Medical Informatics and Technology, Private University for Health Sciences, Innsbruck, Austria; 3 Fowler Kennedy Sport Medicine Clinic, University of Western Ontario, London, ON Canada; 4 Department for Life Quality Studies, University of Bologna, Bologna, Italy; 5 IIa Clinica Ortopedica E Traumatologica, IRCCS Istituto Ortopedico Rizzoli, Bologna, Italy; 6 Central Institute of Orthopedics, Vardhman Mahavir Medical College and Safdarjung Hospital, New Delhi, 110029 India; 7 Clinical Orthopedics, Department of Clinical and Molecular Sciences, Università Politecnica Delle Marche, Ancona, Italy; 8 The Orthopaedic Speciality Clinic, Pune, India

**Keywords:** Anterior cruciate ligament, ACL, Hamstring tendon, Quadriceps tendon, Systematic review, Meta analysis

## Abstract

**Purpose:**

The purpose of this study was to synthesize and quantitatively assess the outcomes of ACL Revision using a quadriceps tendon (QT) graft and to compare them with those of ACL Revisions performed with hamstring tendons (HT) graft.

**Methods:**

A comprehensive search based on the PRISMA protocol was performed across PubMed, Scopus, Embase, and Cochrane Library from inception until February 2022. Clinical studies reporting the outcomes of ACL Revision with QT autograft were included. Subjective and Objective IKDC, Tegner activity level, Lysholm knee score, KOOS score, VAS for pain, knee laxity (KT-1000/2000 arthrometer, Lachman test, and pivot-shift test), and graft failure were assessed. A systematic review and meta-analysis were performed and a quality assessment of the included studies was carried out with the MINORS score.

**Results:**

Seven studies met the selection criteria and were included in the systematic review for the qualitative synthesis of data. A pooled mean of all the variables was provided for the 7 studies, while 3 studies included a control group of ACL Revision with HT and were included in a meta-analysis. A total of 420 participants with a mean age of 28.9 ± 10.5 years and a mean postoperative follow-up of 39.3 ± 16.4 months were assessed. Of these, 277 patients underwent ACL Revision with QT and 143 patients underwent ACL Revision with HT. In the QT group, average graft failure was 9.8% compared to 17.4% in the HT group. KOOS Sport and pivot-shift test showed better postoperative outcomes in QT than HT, although it was not statistically significant (*p* = 0.052).

**Conclusion:**

The QT autograft was associated with an improved trend of rotatory laxity, PROMs and failure rate compared to HT autograft after revision ACL reconstruction. The QT autograft for revision ACL reconstruction is supported by the current literature. It is a viable graft that should be considered for both primary and revision ACL reconstruction.

**Level of evidence:**

Level IV.

## Introduction

The increasing rates of Anterior Cruciate Ligament (ACL) reconstructions lead to an increased burden of failures which will subsequently need revision surgery [1–3]. Revision reconstruction is technically more challenging due to the removal of prior implants, appropriate tunnel positioning, muscle weakness following the primary reconstruction, and higher concomitant injuries [4, 5]. As techniques have improved so have the outcomes following revision surgery, however, inferior results were reported with a revision surgery than with primary ACL surgery [4, 6]. A higher failure rate has been reported with revision ACL reconstruction compared to primary reconstruction, highlighting the clinical relevance of revision surgery for failed ACL reconstruction [1, 7].

In primary ACL reconstruction, hamstring tendon (HT) and bone–patellar tendon–bone (BPTB) autografts are the most commonly used graft, [8]. However, in a revision case where the ipsilateral graft has already been used, it will be difficult to convince the patient to undergo a procedure on the contralateral uninjured limb, for graft harvesting [9].

In recent years, the quadriceps tendon (QT) autograft has gained popularity for ACL reconstruction [10]. The QT graft has been reported to provide a broader base for anatomic insertion of the reconstructed ACL to the tibia [11], has a greater mean cross-sectional area compared to the BPTB and HT [12], and has a greater load to failure than other grafts. Harvesting of QT autograft has lower donor-site morbidity than BPTB and HT harvesting [13] and preservation of muscle strength in knee flexion compared to the HT graft [14]. Despite these potential benefits that can translate into better clinical outcomes, the QT graft has been studied less extensively, particularly in the revision setting. Of the few studies available, some are retrospective reviews of data with relatively small sample sizes that do not allow generalization of the findings [5, 15, 16].

To the best of the authors’ knowledge, no systematic review or meta-analysis is available in the literature on the QT autograft used in revision ACL reconstruction. Therefore, the purpose of this study was to synthesize and qualitatively assess the evidence available currently in the literature on the QT graft for revision ACL reconstruction. The hypothesis was that better functional outcomes and lesser graft failure will be found in the QT group compared to HT graft for revision ACL reconstruction.

## Materials and methods

This meta-analysis followed Preferred Reporting Items for Systematic Reviews and Meta-Analyses (PRISMA) guidelines to identify and extract eligible articles [17] and was registered on the PROSPERO International Prospective Register of Systematic Reviews (ID: CRD42022308299).

### Search strategy

Two independent authors (A.M. and S.D.P.) conducted a comprehensive search across multi-databases (PubMed, Scopus, Embase, and Cochrane Library) and reviewed each article’s title and abstract for studies available until February 2022. The search terms used were (ACL OR “anterior cruciate ligament”) AND (“quad*” OR “QT”) AND (“revision“Z” OR “reoperation”). The full texts of the studies were evaluated when eligibility could not be assessed from the title and abstract. Both of these two authors independently assessed the eligibility of studies and any disagreements were resolved by discussion if disagreement could not be resolved, then the senior author (C.F.) was consulted.

### Inclusion and exclusion criteria

Clinical studies of any study design reporting on outcomes following revision ACLR with QT autograft were considered for inclusion. The following outcomes were looked at in the studies: IKDC score, Tegner activity level, Lysholm knee score, KOOS score, VAS for pain; knee laxity measured with KT-1000/2000 arthrometer, Lachman test, and pivot-shift test. Non-English studies, review articles, non-peer-reviewed studies, surgical techniques, case reports, conference abstracts, biomechanical studies, as well as studies solely focused on primary ACL reconstruction, use of graft other than QT autograft, sample size less than 15 patients, and minimum follow-up less than 2 years were excluded.

### Methodological quality assessment

Quality assessment of the included studies was performed using the methodological index for non-randomized studies (MINORS) criteria (Table 1) [18]. Two authors (A.M. and S.D.P.) independently assessed the quality of each article. The senior author (C.F.) was consulted in case of disagreement.

**Table 1:** MINORS scale to assess study quality

MINORS	Noyes 2006	Garofalo 2006	Haner 2016	Barie 2019	Eggeling 2021	Hunnicut 2021	Supreeth 2022
A clearly stated aim	1	2	2	1	2	2	2
Inclusion of consecutive patients	2	0	2	2	1	1	1
Prospective collection of data	2	0	2	0	0	2	0
Endpoints appropriate to the aim of the study	2	1	2	2	2	2	2
Unbiased assessment of the study endpoint	0	0	0	0	0	0	0
Follow-up period appropriate to the aim of the study	2	2	1	2	1	1	2
Loss to follow up less than 5%	2	0	1	0	1	0	0
Prospective calculation of the study size	0	0	2	0	0	0	0
*Additional criteria in the case of comparative study* An adequate control group			0	0	0		
Contemporary groups			2	2	2		
Baseline equivalence of groups			0	0	0		
Adequate statistical analyses			2	2	2		
Total	11	5	16	11	11	8	7

### Data extraction

Demographic data including age at surgery, sex, and follow-up duration were extracted to provide an overview of the cohort. For each study, the following data were extracted: IKDC score, Tegner activity level, Lysholm knee score, KOOS score, VAS for pain; knee laxity measured with KT-1000/2000 arthrometer, Lachman test, pivot-shift test, pivoting sports, and graft failure rates (Table 2).

**Table 2:** Data summary from the systematic literature search

Authors years	Study design	N° of patients/n° of knees	Outcomes	Results			
				QT		HT	
				Pre-op	Post-op	Pre-op	Post-op
Barié 2019	Retrospective	QT 41	KT-1000 SSD (mm)		1.6 ± 2.0		1.9 ± 2.0
		HT 37	Pivot-shift test (− / + / + + / + + +)				
			(–)		35		19
			( +)		6		11
			(+ +)		0		3
			(+ + +)		0		1
			Tegner Activity level before injuries (median. range)	7(3–10)		9(5–10)	
			Lysholm score		82 ± 14		84 ± 10
			IKDC 2000 subjective evaluation form		79 ± 15		82 ± 12
			Difference in Tegner activity score between preinjury and on follow-up		2 (range 0–6)		2 (range 0–6)
			Pivoting sports (yes/no)		10/29		18 /19
			Patient satisfaction		9 (range 0–10)		9 (range 4–10)
			SLTH (single-leg-triple-hop-test) (> 90%/76–89%/50–75%/ < 50%)		25/6/2/0		25/6/0/0
Eggeling 2021	Retrospective case series	QT 43	VAS pain	3.6 ± 2.5 (0–8)	0.9 ± 1.1 (0–3)	4.1 ± 2.4 (0–10)	1.6 ± 2 (0–9)
		HT 46	Lysholm score	54.9 ± 15.1 (10–77)	85.4 ± 13 (43–100)	51.3 ± 25 (7–77)	83.2 ± 17 (25–100)
			Tegner score	3.2 ± 1.3 (1–6)	5.8 ± 1.8 (3–9)	2.9 ± 1.4 (0–6)	5.6 ± 1.5 (1–9)
			Pivot-shift test (− / + / + + / + + +)				
			(–)	4	41	1	41
			( +)	7	1	13	2
			(+ +)	20	1	17	2
			(+ + +)	12	0	15	1
			Rolimeter SSD Postoperative		1.3 ± 1.3 (0–5)		1.8 ± 2.2 (0–9)
			IKDC score (Postoperative)		83.8 ± 12.2 (37–100)		78.6 ± 16.8 (14–100)
			Failure of revision ACLR		1 (2.3)		8 (17.4)
			KOOS subscale (Postoperative)				
			Symptoms		87 ± 15.3 (50–100)		87 ± 16.2 (25–100)
			Pain		90.2 ± 11.6 (56–100)		88.7 ± 12.6 (36–100)
			ADL		94.1 ± 8.5 (71–100)		94.2 ± 8.7 (59–100)
			Sports/Rec		80.1 ± 20 (30–100)		75.7 ± 19 (25–100)
			Quality of life		62.5 ± 23 (13–94)		58.4 ± 22 (0–88)
			Lachman test grade				
			Absent	0 (0)	39 (90.7)	0 (0)	36 (78.3)
			Grade 1 (2–5 mm)	2 (4.7)	3 (7)	12 (26.7)	3 (6.5)
			Grade 2 (5–10 mm)	31 (72.1)	1 (2.3)	29 (64.4)	7 (15.2)
			Grade 3 (> 10 mm)	10 (23.3)	0 (0)	4 (8.9)	0 (0)
			Donor-site morbidity		2 (4.7)		6 (13)
Häner 2016	Prospective comparative study	QT 25	Lysholm score (Postoperative)		85.8 ± 14.5		77.7 ± 19.6
		HT 26	KOOS Symptoms (Postoperative)				
			Symptoms		77.1 ± 26.5		66.9 ± 30.1
			Pain		83.6 ± 23.6		79.5 ± 22.8
			ADL		91.5 ± 15.9		92.1 ± 12.6
			Sports/Rec		79.0 ± 20.5		62.4 ± 39.2
			Quality of life		60.4 ± 27.2		51.1 ± 29.5
			KT-1000 SSD (mm)	6.4 ± 2.5	2 ± 1.2	5.9 ± 1.6	3 ± 2.9
			IKDC Objective Grading				
			Normal (A)	0	13	0	10
			Nearly Normal (B)	5	5	5	6
			Abnormal (C.)	13	2	14	4
			Severely Abnormal (D)	2	0	0	0
Garofalo 2006	Retrospective	QT 28					
			Pivot-shift test (− / + / + + / + + +)				
			(–)	0	21		
			( +)	6	7		
			(+ +)	13	0		
			(+ + +)	3	0		
			KT-1000 arthrometer side-to-side difference	8.2 ± 3.2	3.1 ± 2.0		
			Tegner activity score	4.2 ± 1.5	6.1 ± 1.4		
			Lysholm score	68 ± 12.5	93.6 ± 8.8		
			Lachman test grade				
			Absent	0	17		
			Grade 1	6	11		
			Grade 2	10	0		
			Grade 3	12	0		
			IKDC Objective Grading				
			Normal (A)	0	5		
			Nearly Normal (B)	4	21		
			Abnormal (C.)	21	2		
			Severely Abnormal (D)	3	0		
Hunnicutt 2021	Retrospective	QT 100 total patient					
		67	IKDC scores Preoperative	54.3 ± 13.0			
		76	IKDC scores Postoperative		82.8 ± 13.8		
		68	KT-1000 SSD at 6-month f/u		1.4 ± 1.6		
		80	Graft failure		11		
			Isokinetic strength: limb symmetry indexes (%)				
			QTriceps LSI at 60/s				
		52	6 months		71.6 ± 19.3		
		40	12 months		81.5 ± 19.3		
			HTstrings LSI at 60/s				
		52	6 months		93.1 ± 19.8		
		40	12 months		100.4 ± 15.4		
			QTriceps LSI at 180o/s				
		53	6 months		76.6 ± 16.4		
		39	12 months		83.9 ± 16.9		
			HTstrings LSI at 180o/s				
		53	6 months		93.7 ± 18.1		
		39	12 months		97.8 ± 17.5		
Noyes 2006	Prospective study	21	KT-2000 arthrometer side-to-side displacement	8.4 ± 3.1	2.1 ± 2.2		
			Pivot-shift test (− / + / + + / + + +)				
			(–)	all knee grade 2 or 3	10		
			( +)		7		
			(+ +)		3		
			(+ + +)		1		
			IKDC knee ligament examination				
			Normal (A)	0	8		
			Nearly Normal (B)	0	9		
			Abnormal (C.)	4	3		
			Severely Abnormal (D)	17	1		
			Overall knee evaluation rating score	54 ± 7	76 ± 16		
Supreeth 2022	Retrospective study	QT 19	Tegner Lysholm functional score		85.35		83.65
		HT 34					

### Statistical analysis

The continuous variables were extracted and analyzed as mean and standard deviation (SD) using an Excel spreadsheet (Microsoft Corporation, Redmond, WA, USA). When median and interquartile ranges were reported, these were converted into mean and SD. If it was not possible to calculate the SD from the available data, the highest SD was used. The categorical variables were reported as sum and percentage over the total.

**Fig. 1:**
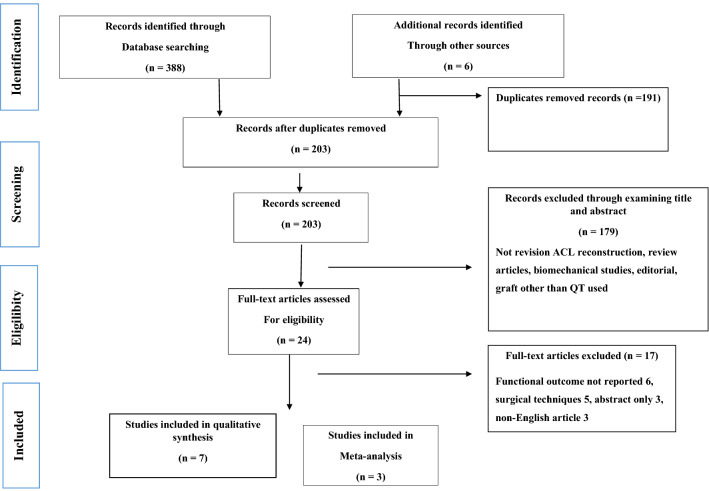
Selection process flow diagram according to the PRISMA guidelines to identify the studies included in the systematic review and the meta-analysis

A meta-mean weighted over the number of patients was computed separately for QT and HT groups for each clinical outcome and laxity measurement. The studies reporting data for both the HT and QT groups were included in a meta-analysis (Fig. 1). The standardized mean difference (SMD) and 95% confidence interval (CI) were calculated for continuous variables referring to the same clinical outcome or laxity measure. The Odds Ratio (OR) and 95% CI were instead computed for the categorical variables. The Higgins’ *I*^2^ statistics were calculated to determine the heterogeneity. The pooled estimates of the effect size were presented as forest plots per each variable. The Mantel–Haenszel random-effects model was used to pool the data if statistically significant heterogeneity was reached; the fixed-effects model was used otherwise. Statistical significance was set at *P* < 0.05. All the analyses were conducted in MedCalc Statistical Software version 19.2.6 (MedCalc Software Ltd, Ostend, Belgium).

## Results

### Search results

For this study, 394 potentially relevant articles were identified in the initial comprehensive search. Following the deletion of duplicate titles, 203 abstracts were yielded for screening. A further 179 articles were excluded based on abstract screening; 24 articles were obtained in full text and the inclusion and exclusion criteria were applied. Seventeen articles were removed after full-text screening due to various reasons. Finally, 7 studies met the selection criterion, but only 3 studies had a control group (HT graft) which underwent metanalysis. All 7 studies were included in the systematic review and for the qualitative synthesis of data (Fig. 1).

### Study design and characteristics

Among the 7 included studies, 1 was a prospective case–control study, 2 were prospective cohorts, and 4 were retrospective studies. All studies reported the use of a single-staged revision procedure except the study by Eggeling et al. [16] where a two-staged revision was reported. According to the MINORS scale, 4 studies were rated as “high quality” (score 11 or more), while 3 as “low quality” (score < 11), mostly due to a lack of unbiased outcomes assessment and prospective size calculation (Table 1).

### Systematic review of outcomes of ACL Revision with QT

A total of 277 patients with a mean age of 30.6 ± 7.1 and a mean postoperative follow-up of 40.2 months underwent ACL Revision with QT and were included in the quantitative synthesis.

#### Patient-reported outcome measures

The Lysholm knee score was reported in 156 patients from 5 studies [9, 15, 16, 19, 20], showing an average postoperative value of 86.4 points. Significant improvement was reported in both the groups with QT and HT grafts. However, pooled averages of Lysholm scores show a higher functional score with QT (86.1) than with HT (82.2) grafts postoperatively. The Subjective IKDC score was reported in patients from 2 studies, showing an average postoperative value of 82.1 points. The KOOS score was reported in 2 studies as well, including 68 patients and showing an average postoperative value of 86.9 points for the Pain subscale, 82.1 for the Symptoms subscale, 92.8 for the ADL subscale, 79.6 for Sports subscale, and 61.5 for Qol subscale. The Subjective IKDC score was reported in 184 patients from 3 studies, showing an average postoperative value of 81.9 points (Table 3).

**Table 3:** Pooled average of the data retrieved from the systematic literature search

Outcome	QT		HT	
	n° of subjects	Pooled average/sum	n° of subjects	Pooled average/sum
Failure of revision ACLR	123	12 (9.8%)	46	8 (17.4%)
Pivoting sports: no/yes	41	29/10	37	19/18
PROMS				
IKDC score (Preoperative)	67	54.3		
IKDC score (Postoperative)	160	82.1	83	80.1
KOOS ADL (Postoperative)	68	93.2	72	93.4
KOOS Pain (Postoperative)	68	87.8	72	85.4
KOOS QoL (Postoperative)	68	61.7	72	55.8
KOOS Sport (Postoperative)	68	79.7	72	70.9
KOOS Symptoms (Postoperative)	68	83.4	72	79.7
Lysholm score (Preoperative)	71	60.1	46	51.3
Lysholm score (Postoperative)	137	86.1	109	82.2
Difference in Tegner Activity score (Pre-injury vs follow-up)	41	2.2	37	2.2
Tegner Activity score (Before injury)	41	6.9	37	8.7
Tegner Activity score (Preoperative)	71	3.6	46	2.9
Tegner Activity score (Postoperative)	71	5.9	46	5.6
VAS pain (Preoperative)	43	3.6	46	4.1
VAS pain (Postoperative)	43	0.9	46	1.6
LAXITY				
KT-1000 SSD (Preoperative)	53	7.4	26	5.9
KT-1000 SSD (Postoperative)	162	1.8	63	2.4
Pivot-shift test preoperative (–/ + / + + / + + +)	71	4/13/33/15	46	1/13/17/15
Pivot-shift test postoperative (–/ + / + + / + + +)	112	97/14/1/0	83	60/13/5/2
Rolimeter SSD (Postoperative)	43	1.3	46	1.8
Single-leg-triple-hop-test (SLTH) (< 50%/50–75/76–89/ > 90%)	41	0/ 2/6/25	37	0/0/6/25

#### Laxity assessment

Clinical measures of laxity used were the KT-1000 arthrometer side-to-side difference (SSD), in 4 studies [5, 9, 15, 19]. Pooled average of postoperative KT-1000 SSD measurement was 1.8 mm for QT and 2.4 mm for HT grafts, and thus, QT grafts showed lesser laxity (Table 3). The manual antero-posterior laxity with the Lachman test was reported in 71 patients from 2 studies, showing negative tests in 56 (78.8%) and grade 1 in 15 (19.7%) of patients. Similarly, the pivot-shift test was reported in 133 patients from 4 studies, showing negative tests in 107 (80.5%) and grade 1 in 21 (15.8%) of patients. Instrumental antero-posterior laxity was reported in 258 patients from 6 studies, showing an average postoperative value of 1.9 mm.

#### Complications and graft failures

A complication was reported in 27 out of 277 patients (9.7%), hypersensitive scars in 5 patients, infection in two cases, donor-site morbidity in two cases, and cyclops syndrome in 1 patient. Graft failure was reported in 15 out of 277 patients, for an overall rate of 5.4%. Failure of revision ACL reconstruction was reported in 4 studies [5, 9, 15, 16]. In the QT group, graft failure was 12 (9.8%) compared to 8 (17.4%) in the HT group.

##### Rehabilitation protocol

The postoperative protocol, reported in all except one study [16], varied among the included studies regarding brace use, weightbearing, and range of motion (Table 4).

**Table 4:** Study designs, technique, and rehabilitation protocol

Authors years	Technique	Rehabilitation Protocol
Noyes 2006	Quadriceps tendon with patellar bone block, Single stage procedure	Brace for 6 weeks with partial weightbearing from 2nd week to full weightbearing at 6^th^ week
Garofalo 2006	Two incision technique with Quadriceps autograft Single stage procedure	Full weightbearing immediate postop. Partial weightbearing with knee brace in extension in cases with meniscus repair
Haner 2016	Ipsilateral bone quadriceps tendon grafts compared with contralateral semitendinosus-gracilis grafts single-stage procedure	Partial weightbearing and full range of motion permitted in both groups. Brace used for 6 weeks
Barie 2019	Quadriceps autograft compared with Hamstring autograft, Single stage procedure	Partial weightbearing for 2 weeks to full weightbearing by 4 weeks in both groups
Hunnicut 2021	all-soft tissue Quadriceps tendon autografts, Single stage procedure	Progressed to full weightbearing with crutches over first 2 weeks, weight training by 6 weeks
Eggeling 2021	double-layered, partial-thickness, soft tissue quadriceps tendon graft (dlQUAD) compared with Hamstring autograft, two-staged procedure in all cases	
Supreeth 2022	Revision cases with Semitendinosus- gracilis autograft, Bone patellar tendon–bone autograft and Quad tendon grafts compared	Partial weightbearing allowed for 2 weeks with general weightbearing at 6 weeks. Supervised sport-specific exercises at 20 weeks

#### Return to sports activity

Return to sports activity was assessed in five of the seven studies [5, 15, 19–21]. Running was allowed at 3 months except in one study [21] where running was allowed at 6 months. In all of the five studies, return to sports was allowed at 6–9 months.

### Meta-analysis of outcomes of ACL Revision with QT versus HT

A total of 109 patients with a mean age at surgery of 34.3 years underwent Revision ACL with QT, while 109 patients with a mean age at surgery of 30.1 years underwent Revision ACL with HT. The follow-up ranged from 24 to 83 months.

#### Patient-reported outcome measures

**Fig. 2:**
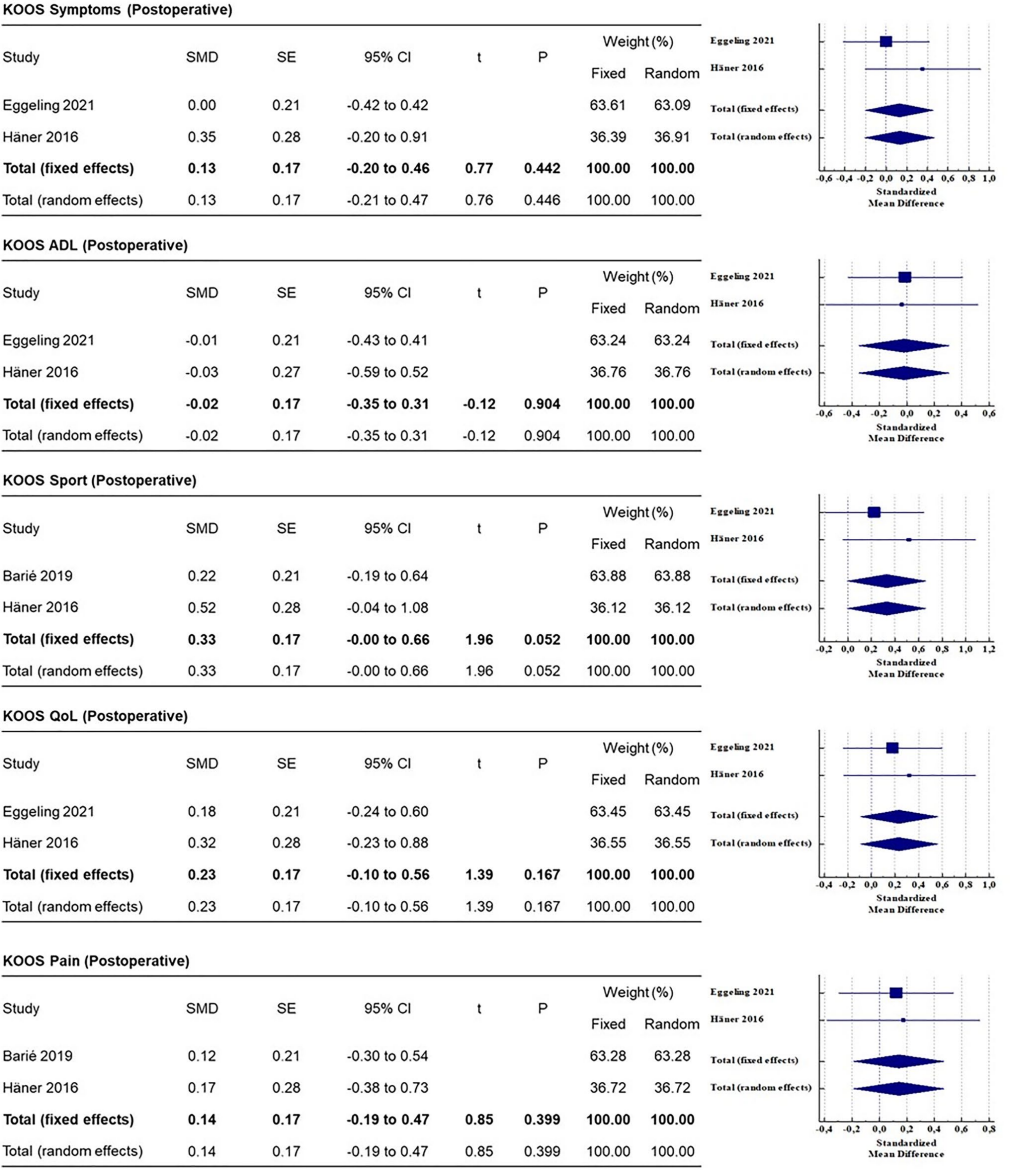
Meta-analysis (part 1), forest plots showing KOOS subscale between HT and QT autografts. The standardized mean difference (SMD) and standard error (SE) or odds ratio (OR) were reported for continuous and categorical variables, respectively. A 95% confidence interval (CI) was reported

**Fig. 3:**
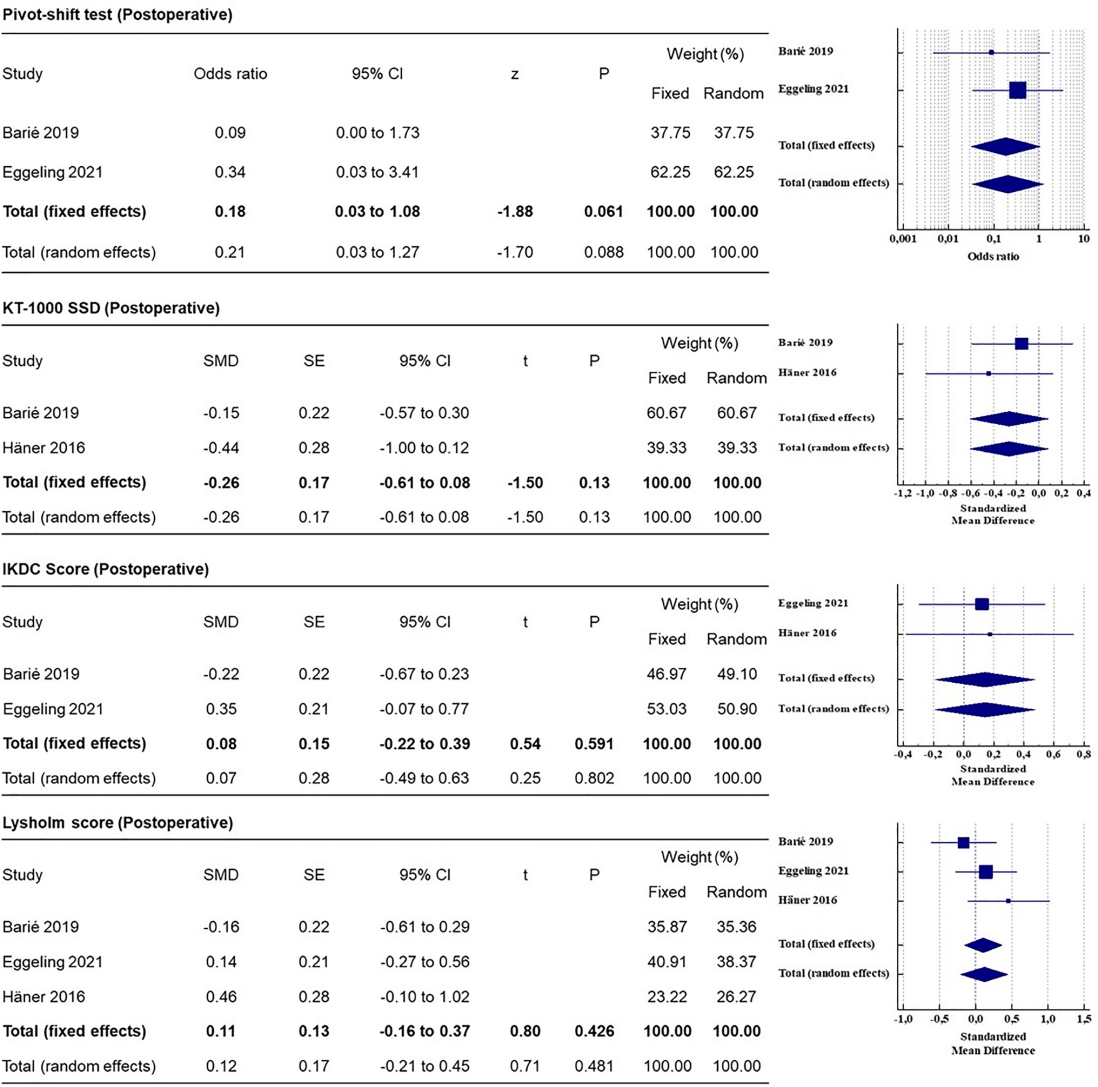
Meta-analysis (part 2), forest plots showing laxity and functional outcomes between QT and HT autografts. The standardized mean difference (SMD) and standard error (SE) or odds ratio (OR) were reported for continuous and categorical variables, respectively. A 95% confidence interval (CI) was reported

The results of the meta-analysis are depicted in Fig. 2 and Fig. 3. The Lysholm knee score used in 3 studies [9, 15, 16] was used for the meta-analysis (on a restricted cohort), which showed that the difference was not statistically significant (*p* > 0.05). The IKDC score, used in 2 studies [15, 16], showed conflicting findings. In the study by Eggeling et al. [16], postoperative IKDC with QT graft was 83.8 ± 12.2 and with HT graft was 78.6 ± 16.8, while in the study by Barie et al. [15], postoperative IKDC with QT graft was 79 ± 15 and with HT graft was 82 ± 12 (Table 2).

The KOOS scale was used in 2 studies [9, 16]. Among the five subscales of the KOOS, pooled average scores were higher for the QT graft for QoL (Quality of life), Sport and Symptom subscales (Table 3). KOOS Sport showed an SMD = 0.33 [0.00–0.66] (n.s., Fig. 2). The KOOS were similar in the ADL and Pain subscales. The Tegner Activity scale was used in 3 studies [15, 16, 19]. The pooled average postoperative Tegner activity levels were higher for QT than for HT (5.9 vs 5.6, n.s.). In the study by Eggeling et al. [16], the postoperative VAS score for pain significantly improved in the QT group compared to the HT group (*p* < 0.05).

#### Laxity assessment

Postoperative KT-1000 SSD measurement showed lower laxity for QT than HT, although it was not significant (n.s., Fig. 3). Similarly, lower (non-significant) laxity was found in Rollimeter SSD in the QT group compared to the HT group [16] and lower (non-significant) pivoting was reported in the QT group compared to the HT group [15, 16].

## Discussion

The most important findings of this study are that the quadriceps tendon graft has good results in revision ACL setting, being similar to the results of ACL revision with hamstring tendon grafts and that there is a trend of better results for certain outcome measures such as the Lysholm knee score, KOOS subscales for Quality of life, Sports activity and symptoms, the Tegner activity levels, and VAS for pain postoperatively. The QT group was also associated with lesser postoperative laxity, quantified using KT-1000 SSD, pivot-shift test, Lachman test, and Rollimeter. Moreover, QT autograft had lesser failure compared to HT graft for revision ACL reconstruction. However, the meta-analysis showed no significant differences between the group for all the variables.

Various studies found equal or better outcomes with QT graft than HT and BPTB autograft in primary ACL reconstruction [13, 14, 22–24]. Runner et al. [23] and Cavaignac et al. [22] compared the functional outcomes between QT and HT groups, and they found better results in the QT group. Similarly, a recent meta-analysis reported higher functional scores in the QT than in the HT group [13]. The findings of the previous studies are similar to the current study; however, these previous studies focused on primary ACL reconstruction, whereas, in the current metanalysis, only revision studies were included. In a recent revision ACL study, the QT group showed small increases in IKDC scores and Tegner activity levels than the HT group [16]. Similarly, in another included study, authors found higher Lysholm, IKDC, and KOOS scores in the QT group compared to the HT group [9]. This suggests that QT autograft is a viable option in both the primary and revision ACL reconstruction than the HT autograft.

In the present study, the QT graft was associated with lesser postoperative laxity compared to the HT graft. In the QT group, no cases of grade 3 laxity were found. Although both graft choices give good results with respect to postoperative laxity, the quadriceps tendon graft seems to edge out the hamstring graft. Lesser postoperative laxity may be a possible reason for better postoperative KOOS sports subscale in the QT group than in the HT group. Similar findings were reported by Cavaignac et al. [22] in their primary ACL reconstruction. They found lower pivot-shift grade, Lachman grade, and side-to-side difference measured by KT-1000 arthrometer in the QT group than HT group. In another study, the QT graft showed lesser laxity on pivot-shift testing and also lower failure rates when compared to the HT graft, in the setting of primary ACL reconstruction surgery [25]. The present study suggests lesser postoperative laxity in the QT group than in the HT group and these findings are similar to previous literature.

In the current study, Tegner activity was better in the QT group than in the HT group (5.9 vs. 5.6). Similarly, KOOS sport was also better in the QT group than in the HT group (79.7 vs. 70.9). These findings indicate a better return to sports after revision ACL reconstruction with QT autograft. In revision ACL reconstruction, majority of surgeons prolong the time for a return to sports. This fact was also verified in the current study where return to sporting activity was allowed at around 6–9 months in the 5 studies [5, 15, 19–21]. Only the study by Barie et al. [15] compared sporting activity participation postoperatively between the QT and HT groups. They found that the proportion of patients participating postoperatively in pivoting sports was significantly higher in the HT group. However, this could be partly explained by the fact that the HT group had a higher Tegner activity level preoperatively than the QT group which would reflect not only better knee health among this group but would also reflect a better overall general physical condition among this group which would influence postoperative rehabilitation and level of activity attained.

The selection of graft is an important factor in revision ACL reconstruction as the rate of graft failure can be directly correlated with the type of graft. Higher graft failure was reported in revision ACL reconstruction with allograft compared to autograft [1, 26]. QT autograft has good anatomical characteristics with respect to graft volume, graft thickness, and graft length; these characteristics are comparable to HT and BPTB autografts [23, 24, 27]. Previous studies showed superior biomechanical properties with QT compared to BPTB with respect to load to failure, strain at failure, and Young’s modulus of elasticity [28, 29]. In the present meta-analysis, a lower graft failure rate was found in the QT group than in the HT group. Eggeking et al. [16] reported significantly higher failures with HT graft than with QT (1 QT failure compared to 8 HT graft failures). Similarly, in a recent meta-analysis, graft failure was significantly higher in the HT group than QT group for primary ACL reconstruction [25].

Despite these promising biomechanical and clinical studies, QT autografts are not widely used. One of the major factors responsible for its lesser use is historical harvesting techniques, where extensive dissection of extensor apparatus leads to quadriceps weakness and graft harvested by older techniques was biomechanically weaker and associated with residual rotatory knee laxity [29]. Moreover, the paucity of long-term clinical trials and large cohort studies makes the QT autograft a difficult choice for surgeons, who are more convenient with conventional BPTB and HT considering their satisfactory long-term results. However, with the increasing number of revision ACLs and the highly active patient population, it is necessary to study other graft options beyond the conventional HT and BPTB. Recently improvements in harvest techniques allow the surgeon to reliably yield a robust volume of QT graft without hampering the quadriceps strength and lesser donor-site morbidity [3, 8, 13, 30, 31]. The finding of this meta-analysis is quite promising for surgeons who are dealing with revision ACL reconstruction and are not sure about graft choice. To the best of the authors’ knowledge, this is the first systematic review and meta-analysis on revision ACL reconstruction using QT autograft.

The present study has some limitations. Revision ACL reconstruction is a less frequently performed procedure than primary reconstruction. Therefore, the authors were limited in the current study by the paucity of studies with high levels of evidence. The available studies were mostly retrospective with limited sample sizes and there was only one prospective comparative study available on revision ACL surgery comparing quadriceps and hamstring tendon grafts. Further hurdles were the non-uniformity of the techniques used heterogeneous nature of the tools and scores used for reporting outcomes. High-quality randomized controlled trials or large cohort prospective studies with homogeneous populations, especially regarding surgical techniques and outcome scores, could provide better evidence for the use of QT autograft for revision ACL reconstruction.

The clinical relevance of the present study lies in the fact that the QT autograft is the least studied and least used graft compared to other grafts, especially for revision ACL reconstruction. Many surgeons do not even consider the QT as a possible graft option when discussing with the patients. However, it is a very suitable and versatile graft option for revision ACL reconstruction with distinct advantages. The contribution of this study to the existing literature is meaningful as it delineates the deficiencies in current literature and shows that QT grafts are a viable graft choice in primary and revision situations.

## Conclusion

The QT autograft was associated with an improved trend of rotatory laxity, PROMs, and failure rate compared to HT autograft after revision ACL reconstruction. The QT autograft for revision ACL reconstruction is supported by the current literature. It is a viable graft that should be considered for both primary and revision ACL reconstruction.

## Data Availability

The datasets generated and analyzed during the current study are available from the corresponding author on reasonable request.
